# Malignant Peripheral Nerve Sheath Tumors Masking as Ewing Sarcoma/Primitive Neuroectodermal Tumors

**DOI:** 10.4021/wjon661w

**Published:** 2013-07-15

**Authors:** Marwan Shaikh, Fauzia Rana

**Affiliations:** a University of Florida College of Medicine Jacksonville, 7651 Gate Parkway #1211, Jacksonville, FL 32256, USA; b Division of Hematology and Medical Oncology, University of Florida College of Medicine Jacksonville, UF Hematology/Oncology at Pavilion, 4th Floor North, Pavilion, 555 West 8th Street, Jacksonville, FL 32209, USA

**Keywords:** MPNST, Malignant, Peripheral, Nerve, Sheath, Sarcoma, Ewing’s, Primitive, Neuroectodermal

## Abstract

Malignant peripheral nerve tumors, a small subset of soft tissue sarcomas, provide a unique diagnostic challenge. Although they may arise from peripheral nerves or from cells associated with nerve sheaths, malignant peripheral nerve tumors often present with diverse immunohistochemical features similar to those of other tumors. These features make MPNSTs difficult to diagnose and classify. We present a case of a 26-year-old female presenting with a rapidly growing soft tissue mass. The mass was excised and immunohistological staining suggested a Ewing’s sarcoma/Primitive neuroectodermal tumor. Confirmational studies did not confirm this diagnosis and upon further review, the diagnosis was changed to a malignant peripheral nerve sheath tumor. We reviewed this case in the setting of the reported literature concerning MPNSTs with focus on the epidemiologic, diagnostic, and immunohistologic features that distinguish this tumor from other similar malignancies.

## Introduction

Malignant peripheral nerve sheath tumors (MPNST) comprise a small subset of only 5-10% of soft tissue sarcomas [1, 2]. While the exact incidence of these tumors is unknown, Ducatman et al found an incidence in their general population of about 0.001% from 1950 - 1982. During a similar time frame, they also found a 4.6% incidence of MPNST among 1124 Neurofibromatosis type 1 patients in their clinic [3]. MPNSTs arise from peripheral nerves or from other cells associated with the nerve sheath such as Schwann cells, perineural cells, and fibroblasts. Each of these cell types has their own morphologic features distinguishing it from the others. As a result, the histological presentations of MPNST vary greatly and provide a unique diagnostic challenge. Per our review, they are only a handful of reports showing MPNSTs with areas of primitive neuroepithelial differentiation. They are also few reports of Ewing’s Sarcoma/Primitive Neuroectodermal Tumors (ES/PNET) mimicking MPNST. The histochemical stains that distinguish these tumors are not always consistent and present with different sensitivities and specificities, thus expanding the differential diagnosis and making it difficult to arrive at a specific diagnosis. Nonetheless, the importance of making an accurate diagnosis is crucial in providing insight to the patient’s prognosis and treatment. Furthermore, while radiotherapy has resulted in great advances in tumor reduction and improved prognosis, their use, unfortunately, carries the risk of secondary tumor formation years later. We present a case of an MPNST resulting from radiotherapy and presenting with features suggestive of an ES/PNET.

## Case Report

**Figure 1:**
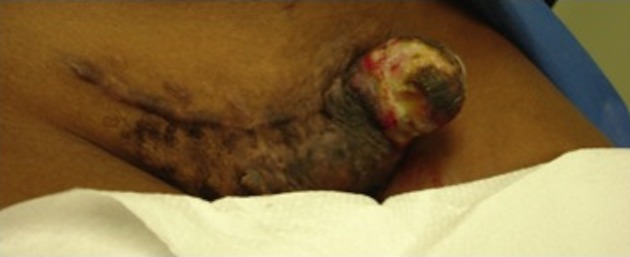
Gross unresected tumor on lateral side of C-section incision.

A 26-year-old African American female presented with a progressively enlarging left inguinal mass for three months. The mass has been more painful and now presented with multiple areas of hemorrhage. At age 17, the patient underwent an uncomplicated pregnancy and C-section. Her C-section incision was complicated with keloid formation for which she underwent two doses of radiation. She remained asymptomatic up until she noticed swelling three months prior. She did not report any other trauma and her only other medical history was significant for a tubal ligation and appendectomy. She had no family history of previous cancer and quit smoking three months prior. Her review of systems was negative except for recent fever and chills. Her vitals were stable and her physical exam was normal except for the inguinal mass and slightly palpable bilateral inguinal lymphadenopathy. The mass protruded through the skin, was very tender to palpation, and expressed multiple areas of hemorrhage. It spanned about five centimeters in diameter along the left lateral aspect of a transverse C-section incision (Fig. 1). Her laboratory data was normal except for a microcytic anemia.

**Figure 2:**
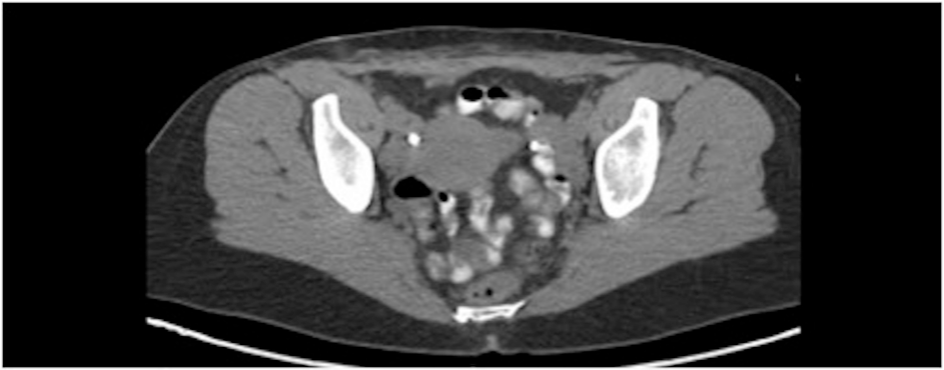
Pelvic CT with abdominal wall soft tissue stranding after tumor resection.

**Figure 3:**
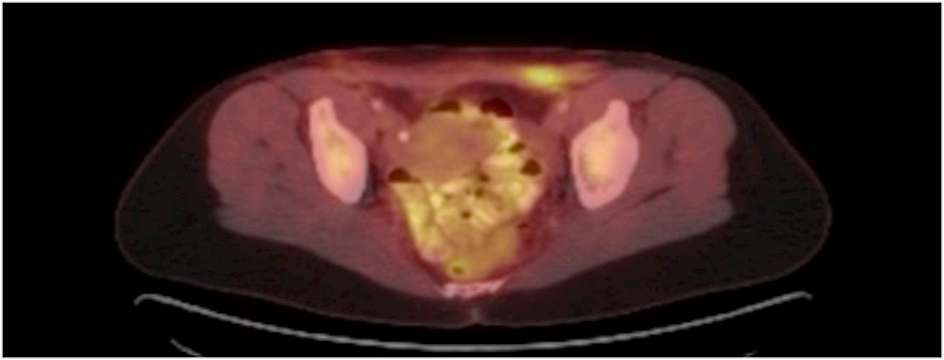
PET CT with hypermetabolic activity with abdominal wall soft tissue stranding and pelvic/inguinal hypermetabolic lymphadenopathy.

The patient initially underwent resection of the mass and given that a sarcoma was suspected, her wound was left open while tumor margins were obtained. The posterior tumor margin returned positive at three millimeters and a reexcision with subsequent wound closer was performed. The initial biopsy report suggested Ewing’s sarcoma/Primitive Neuroectodermal Tumor with findings concerning for melanoma. A EWSR-1 gene rearrangement and BRAF V600E mutation were then sent to confirm ES/PNET and melanoma respectively. A PET-CT revealed hypermetabolic activity with soft tissue stranding in the subcutaneous region of the anterior pelvis, bilateral pelvic and inguinal hypermetabolic lymph nodes, and a four-millimeter left apical lung nodule (Fig. 2, 3). These findings were concerning for a metabolically active neoplasm.

The patient subsequently completed two cycles of chemotherapy with cyclophosphamide, doxorubicin and vincristine alternating with ifosfamide and etoposide. By this time, the confirmatory genetic studies returned negative, thus questioning the original diagnosis. The case was then referred to another expert pathologist at Brigham and Women’s hospital in Boston who suggested MPNST. The patient underwent localized radiotherapy with follow up MRI showing minimal soft tissue stranding, resolving postsurgical change, and no definite residual or recurrent tumor. Previously identified inguinal and external iliac lymph nodes were no longer evident. On follow up she finished radiotherapy and returned back to work.

**Figure 4:**
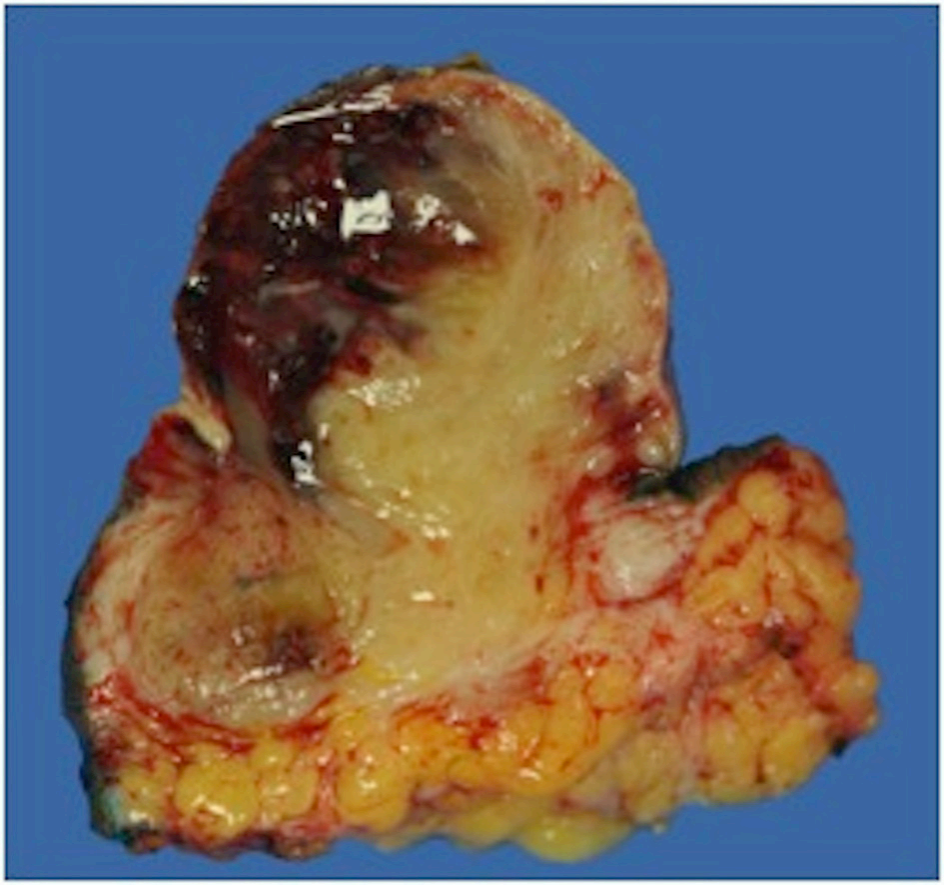
Gross resected tumor with fatty and myxoidal features and focal hemorrhage.

**Figure 5:**
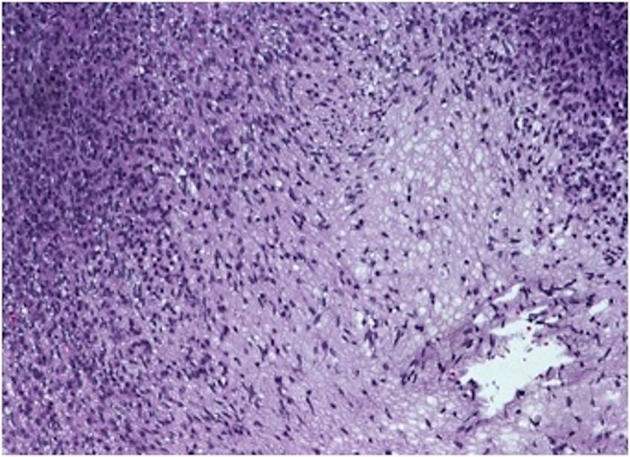
20 × view with spindle cells and areas with loose and dense matrix of small round blue cells.

**Figure 6:**
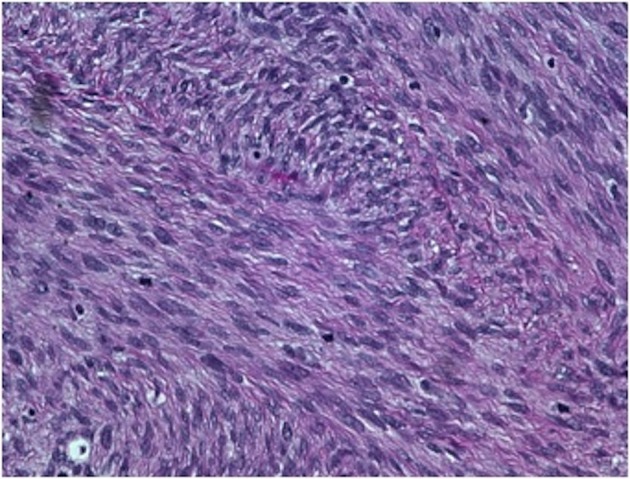
40 × view with small round blue cells as well as spindle cells arranged in a wavy fasicular pattern suggestive of neural origin.

On gross examination, the biopsy specimen had a shiny appearance suggesting myxoidal features along with other areas of hemorrhage (Fig. 4). On histopathological examination (Fig. 5, 6), the specimen again revealed myxoidal features with small round blue cells and other areas of spindle cells arranged in a wavy streamline pattern. There were also areas of high mitotic activity. Histochemically, the specimen stained positive for vimentin and CD99. It stained diffusely positive for S100 and had patchy staining for NSE. It stained negatively for CD31, CD34, desmin, melan-A, SMA, pan-keratin, EMA, CD45, CD56, chromogranin, and synaptophysin.

## Discussion

The importance of correctly diagnosing a MPNST cannot be understated. While representing a small proportion of soft tissue tumors, these tumors present similarly and overlap histopathologically and histochemically with other tumors of their class. Our report provides as example of the difficulty in differentiating these tumors. An accurate diagnosis, therefore, should be based on clinical, radiographic, pathologic findings and genetic testing.

Clinically and radiographically, findings may suggest a soft tissue sarcoma and if attached to a peripheral nerve, a peripheral nerve sheath tumor is the likely diagnosis. This situation, however, is not always the case, as described by Mitchell et al who report a case of an ES initially thought to be a MPNST due to its association with an L4 nerve root [4]. Mohan et al also reported two similar cases of ES: one arising from an ulnar nerve and another from the radial and posterior interosseous nerve [5]. Furthermore, the nerve of origin of a MPNST may never be found. Out of 31 MPNST patients, Nambison et al report that an associated nerve could not be identified in 61% [6]. Another report by Bilgic et al suggests that among NF1 patients with MPNST, the nerve origin could be surgically indentified in only 45-56% of cases [7]. In our case, a soft tissue sarcoma was suspected during surgery, but no associated nerve was identified.

Generally speaking, CD99 and S100 have been routinely associated with ES and MPNST respectively. Making a diagnosis based solely on these stains, however, is unreliable. In a series of 16 MPNST (10 primary, 6 recurrent), Zhu et al found 75% of specimens with positive S100 staining [8]. Olsen et al also report a cluster analysis of the prevalence of 22 antibodies in synovial sarcoma (n = 23), MPNST (n = 23) and ES (n = 27). Their studies revealed significant overlap of many of the antibodies, including CD99 and S100. For example, cytosolic CD99 stained strongly positive in 70% of synovial sarcomas. It also stained strongly positive in 43% of MPNST and 93% of ES. Membranous CD99 staining was more specific (85% specificity, 78% sensitivity) to ES with no staining for MPNST and 26% for synovial sarcoma. These findings may suggest that membranous CD99 can differentiate between MPNST and ES. The study also found S100 to have a specificity of 54% and a sensitivity of 57% among its MPNST cases; further supporting the notion that it is an imperfect test. Finally, the paper found that if multiple stains were used together, the overall specificity improved, namely S100 and nestin (specificity 100%, sensitivity 48%) for MPNST and CD99 and Fli-1 (specificity 96%, sensitivity 56%) for ES. They found nestin to be the best marker for MPNST (specificity 95%, sensitivity 78%) [9]. Our case, unfortunately, did not include stains for Fli-1 and nestin, but did stain positive for vimentin, which per Olsen et al also had a low specificity among their population of MPNST [9]. We must also consider that these findings by Olsen et al are based on the fact that all but one of their MPNST cases were spindle cell type just like our case. Also confounding the diagnosis is that like MPNST, as in our case, PNET tumors can also stain positive for NSE [1].

The question then arises as to how to accurately reach a diagnosis in the setting of multiple overlapping stains. The most reliable ways to confirm ES are with reverse transcriptase-polymerase chain reaction (RT-PCR) and fluorescent in situ hybridization (FISH) studies demonstrating t(11;22) translocation involved in expression of a EWS/FLI-1 fusion transcription factor. Mitchell et al and Mohan et al confirmed their ES diagnoses with these tests, respectively [4, 5].

On microscopic examination, ES/PNET and MPNST also show similar patterns. MPNSTs themselves have variable differentiation, such as rhabdomyoblastic differentiation, glandular malignant differentiation, malignant triton tumor, epithelioid malignant shwannoma, and superficial epithelioid differentiation. In general, however, MPNSTs are marked by intermixed dense cellular fasicles and myxoid regions (marbleized pattern) with round or fusiform cells [2]. There may also be varying degrees of mitosis, necrosis, and calcification [10]. Similarly, ES/PNETs show round small cells arranged in sheets, lobules, and rosettes [1]. In a series of 120 MPNST cases, Ducatman et al notes that only a small percentage of the MPNST cases showed typical Schwannian differentiation including cellular palisading, heterologous elements, and a wavy, cytoplasmic, nuclear configuration [3].

The treatment of choice for MPNST is surgical resection with clear margins. This may be difficult to achieve depending on the location such as in the spine. The current recommendation also includes adjuvant radiotherapy for localized control. There are also some reports of radiotherapy without surgical resection, but this is still controversial and needs further study. Chemotherapy has an even less role in MPNST. While chemotherapy is generally based on that of other soft tissue sarcomas, its efficacy is still needs further investigation [8]. In contrast, chemotherapy plays an important role in treatment of ES/PNET; thus further necessitating an accurate diagnosis [1, 4, 5].

The other interesting finding about radiotherapy is that it, like in our case, may also increase the risk of future MPNST. Ducatman et al also cites a small sample of 13 postradiation MPNST cases among 120 overall cases. They note a latency period averaging 16.9 years between initial radiation and tumor diagnosis [3].

In terms of prognosis, there are a number of tumor features and characteristics to consider. While the prognosis of MPNST is generally thought to be worse with NF1, there may be conflicting evidence [2, 11]. If treated aggressively, MPNST may have a similar prognosis to other deep and high-grade soft tissue sarcomas [11]. As might be expected, Zou et al found an elevated risk of local recurrence in their subset of patients with positive margins who had complete resection [12]. The rate of recurrence is also relatively high with rates from 20-40% and five-year survival ranging from 34-52% [11]. Ducatman et al notes that of 62 patients with NF1, 28 (45%) had at least one recurrence with mean disease free survival of 13.3 months (range 2 - 73 months). Of the 58 patients without NF1, 22 (38%) had at least one recurrence with a mean disease free survival of 32.2 months (range 3 - 102 months). Metastases occurred in 24 of 62 (39%) MPNST patients with NF1 and 9 of 58 (16%) MPNST patients without NF1. The mean interval to diagnosis was 19.3 months (range 0 - 77 months) and 75.1 months (range 13 - 213 months) respectively. The primary location for metastases was the lung, followed by soft tissue, and then bone [3].

There also appears to be an increase in survival (P = 0.001) and less recurrence (P = 0.0001) in patients at least 30 years old than in younger patients [13]. Of the 28 patients in the study by Wanebo et al, the median disease-free survival and median survival time for older patients was 204 months and 216 months, respectively, compared to 2.5 months and 13 months in patients under age 30, respectively [13].

### Conclusion

MPNSTs are a rare subset of soft tissue sarcomas that have clinical, radiographic, and histopatholoigic features similar to other tumors such as ES/PNET. Accurate diagnosis based on pathological and histochemical staining can be challenging yet very important due to their prognostic and treatment implications.
